# Variations of the extrapsoas course of the lumbar plexus with implications for the lateral transpsoas approach to the lumbar spine: a cadaveric study

**DOI:** 10.1007/s00701-024-06216-6

**Published:** 2024-08-02

**Authors:** Michal Benes, Michal Zido, Petr Machac, Radek Kaiser, Anhelina Khadanovich, Simona Nemcova, Vojtech Kunc, David Kachlik

**Affiliations:** 1 Department of Anatomy, Second Faculty of Medicine, Charles University, Prague, Czech Republic; 2 Center for Endoscopic, Surgical and Clinical Anatomy (CESKA), Second Faculty of Medicine, Charles University, Prague, Czech Republic; 3 Department of Neurology, Third Faculty of Medicine, Charles University and University Hospital Kralovske Vinohrady, Prague, Czech Republic; 4 Department of Traumatology, University of Szeged, Szeged, Hungary; 5 Spinal Surgery Unit, Oxford University Hospitals NHS Foundation Trust, Oxford, UK; 6 Research Centre, Faculty of Health Studies, Jan Evangelista Purkyne University in Usti Nad Labem, Usti Nad Labem, Czech Republic; 7 Department of Health Care Studies, College of Polytechnics, Jihlava, Czech Republic

**Keywords:** Genitofemoral nerve, Femoral nerve, Iliohypogastric nerve, Ilioinguinal nerve, Lateral transpsoas approach, Lateral femoral cutaneous nerve, Lumbar plexus, Obturator nerve

## Abstract

**Background:**

Together with an increased interest in minimally invasive lateral transpsoas approach to the lumbar spine goes a demand for detailed anatomical descriptions of the lumbar plexus. Although definitions of safe zones and essential descriptions of topographical anatomy have been presented in several studies, the existing literature expects standard appearance of the neural structures. Therefore, the aim of this study was to investigate the variability of the extrapsoas portion of the lumbar plexus in regard to the lateral transpsoas approach.

**Methods:**

A total of 260 lumbar regions from embalmed cadavers were utilized in this study. The specimens were dissected as per protocol and all nerves from the lumbar plexus were morphologically evaluated.

**Results:**

The most common variation of the iliohypogastric and ilioinguinal nerves was fusion of these two nerves (9.6%). Nearly in the half of the cases (48.1%) the genitofemoral nerve left the psoas major muscle already divided into the femoral and genital branches. The lateral femoral cutaneous nerve was the least variable one as it resembled its normal morphology in 95.0% of cases. Regarding the variant origins of the femoral nerve, there was a low formation outside the psoas major muscle in 3.8% of cases. The obturator nerve was not variable at its emergence point but frequently branched (40.4%) before entering the obturator canal. In addition to the proper femoral and obturator nerves, accessory nerves were present in 12.3% and 9.2% of cases, respectively.

**Conclusion:**

Nerves of the lumbar plexus frequently show atypical anatomy outside the psoas major muscle. The presented study provides a compendious information source of the possibly encountered neural variations during retroperitoneal access to different segments of the lumbar spine.

## Introduction

The lateral transpsoas approach is a minimally invasive access to the lumbar spine (lumbar part of the vertebral column) and adjacent structures. Alternatively, this approach is covered under procedures for intervertebral body fusions known as extreme lateral interbody fusion (XLIF) or direct lateral interbody fusion (DLIF). This approach has become a preferable option over the past years due to a shorter hospital stay, smaller incision extent, superior blood loss control, and especially decreased postoperative pain. However, postoperative complications have been reported with relatively high incidence [5, 17]. Neural deficit poses a frequent complication since the approach pathway goes through the retroperitoneum, where the nerves of the lumbar plexus are located, followed by splitting of the psoas major muscle, being in the nearest proximity to the lumbar plexus itself.

The neural structures are put at risk both during the early stages of superficial dissection at the initial incision site and adipose dissection of the retroperitoneum, as well as during deep splitting of the psoas major muscle. Although real-time nerve monitoring is generally used to identify the morphological relations of the neural structures within the psoas major muscle, such monitoring fails in detection of nerves running outside of the psoas major muscle [4]. Thus, precise knowledge of the surgical anatomy is highly recommended while performing the lateral transpsoas approach together with meticulous dissection skills due to the topographical complexity of the lumbar plexus.

Exploration of medical databases showed that the formation of the lumbar plexus and its relationship to the psoas major muscle has been reasonably documented, including definitions of safe intervals relevant to the approach [3, 5, 7, 14]. Similarly, the trajectories of the main branches of the lumbar plexus within the retroperitoneum have been described [4]. Nonetheless, all previous reports expect standard morphological appearances of the neural structures. Therefore, our objective was to conduct a descriptive study on the variational anatomy of the extrapsoas course of the peripheral nerves emerging from the lumbar plexus in a considerable sample. This study aims to outline the potentially encountered variations in order to prevent neural injury during the lateral transpsoas approach, which may be severe and potentially devastating.

## Material and methods

This study received approval from the Ethics Committee for Multi-Centric Trials of the Motol University Hospital and Second Faculty of Medicine, Charles University in Prague (No. EK-1107/22), and was conducted in adherence to the tenets of the 1964 Declaration of Helsinki and its later amendments.

### Specimens

The anatomical investigations were performed on a total of 260 formalin-preserved lumbar regions obtained from donation programs of the Institute of Anatomy, First Faculty of Medicine; Department of Anatomy, Second Faculty of Medicine; and Department of Anatomy, Third Faculty of Medicine, Charles University in Prague, Czech Republic. All specimens belonged to the Central European ethnicity. The sample comprised 141 right and 119 left lumbar regions. Sex was not recorded because the majority of 138 lower limbs with attached sacral and lumbar spine were isolated from the corpses. Additionally, 62 whole bodies were dissected bilaterally, giving another 124 lumbar regions.

### Dissection protocol

The lumbar plexus was approached through a cross-shaped incision of the ventrolateral abdominal wall with all four flaps reflected tangentially. This step was modified in the isolated lumbar regions according to the amount of attached soft tissues. All intraperitoneal visceral organs were removed and the retroperitoneum was accessed by meticulous dissection of the posterior wall of the peritoneal cavity with special attention paid to the neural structures. Simultaneously, the fasciae and the adipose capsule of the kidneys were carefully extracted and the kidneys were retracted to visualize the nerves properly. The components of the lumbar plexus were visually identified exiting the psoas major muscle and were closely studied. All dissections were performed by three authors with experience in anatomical dissection courses.

### Morphological evaluation and measurements

Each nerve exiting the psoas major muscle was traced to its entrance into the abdominal wall or exit from the pelvic cavity, and was evaluated for the presence of any morphological variations. All unclear cases were photographed and subsequently discussed with other authors to assure objectivity. Moreover, the thickness of each nerve was measured right after its exit from the psoas major muscle using a digital caliper with an accuracy of ± 0.2 mm (Extol Craft, Czech Republic). Eventually, the same tool was used to measure distances between emerging and branching points of a particular nerve when applicable.

### Statistical analysis

The morphometric data are presented as mean values with standard deviation. The frequencies of individual variations are reported as raw numbers and percentages. For comparison of the proportions between right and left side, the Chi-square was used. The level of statistical significance was set at *P* < 0.05. The statistical analysis was performed using GraphPad Prism v. 9.5.1 (GraphPad Software, USA).

## Results

All findings are reported relevant to each nerve below.

### Iliohypogastric nerve

**Fig. 1:**
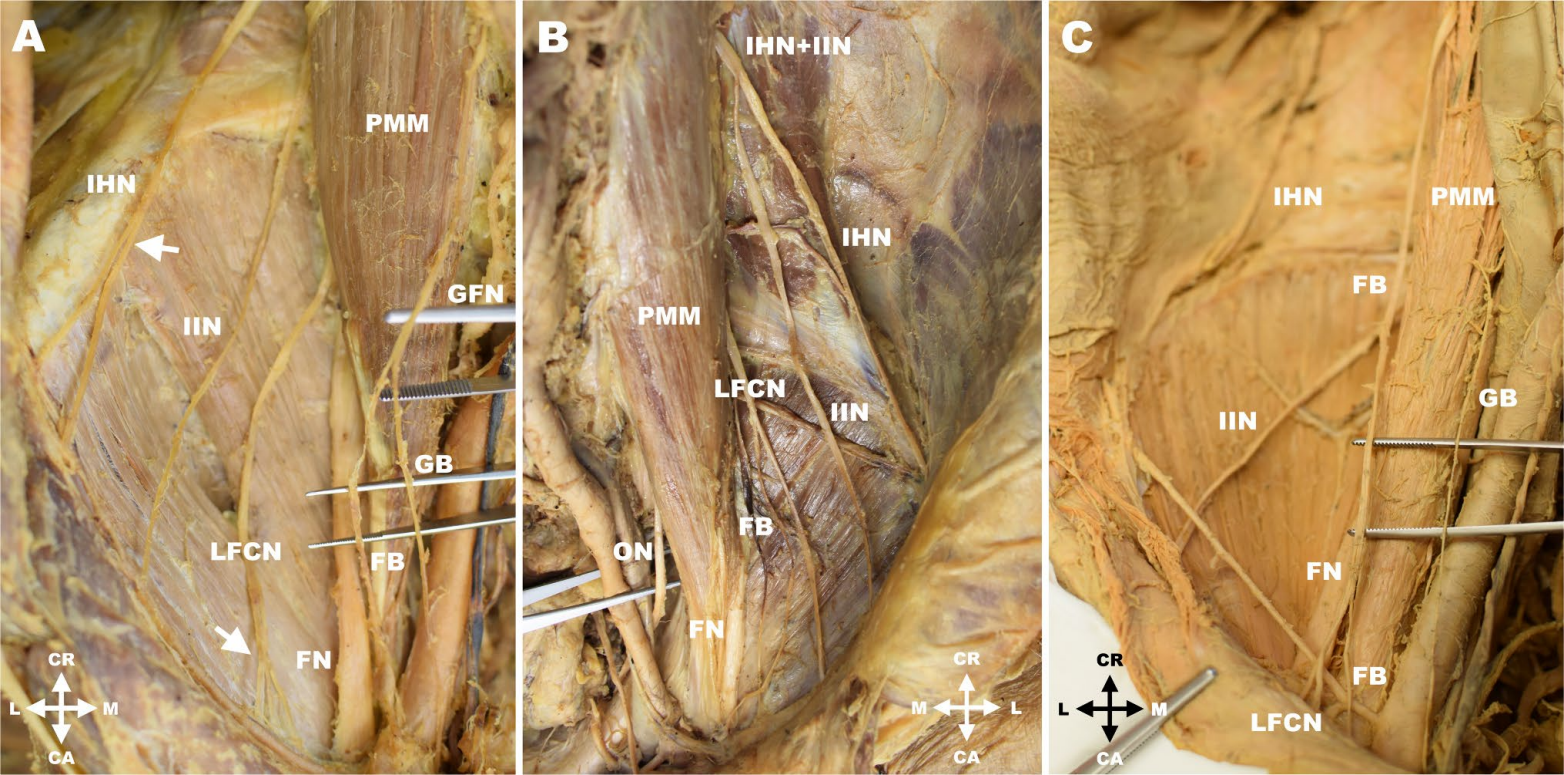
**A**. Right lumbar region with a high branching of the iliohypogastric nerve (IHN) and lateral femoral cutaneous nerve (LFCN). **B**. Left lumbar region with fused iliohypogastric and ilioinguinal nerves (IHN + IIN), and origin of the femoral branch of the genitofemoral nerve (FB) from a common trunk with the lateral femoral cutaneous nerve (LCFN). **C**. Right lumbar region with separate exits of the femoral (FB) and genital (GB) branches of the genitofemoral neve from the psoas major muscle, and unusual origin of the lateral femoral cutaneous nerve (LFCN) from a common trunk with the femoral branch of the genitofemoral nerve (FB). Legend: CA = caudal; CR = cranial; FB = femoral branch of genitofemoral nerve; FN = femoral nerve; GB = genital branch of genitofemoral nerve; GFN = genitofemoral nerve; IHN = iliohypogastric nerve; IIN = ilioinguinal nerve; L = lateral; LFCN = lateral femoral cutaneous nerve; M = medial; ON = obturator nerve; PMM = psoas major muscle. White arrows show branching points of distinct nerves

The iliohypogastric nerve left the psoas major muscle with a diameter of 2.5 ± 0.7 (1.0–4.3) mm. In the majority of cases the nerve ran through the posterior portion of the psoas major muscle and coursed latero-caudally laying on the quadratus lumborum and transversus abdominis muscles. In 198 cases (76.2%) the nerve divided into two branches (anterior and lateral) before entering the dorsolateral abdominal wall (Fig. 1A), and in six cases of these (3.0% from the 198 cases) it featured a high division shortly after its exit from the psoas major muscle. Only in 34 cases (13.1%) the iliohypogastric nerve entered the abdominal wall prior to its division. In another 25 cases (9.6%) the iliohypogastric nerve was fused with the ilioinguinal nerve, and this common trunk either divided to the proper iliohypogastric and ilioinguinal nerves (13 cases; 5.0%) (Fig. 1B) or pierced the abdominal wall without division (12 cases; 4.6%). In the remaining three cases (1.2%) a communicating branch was found between the iliohypogastric nerve and the subcostal nerve. Detailed results are summarized in Table 1.

**Table 1:** Proportions of the individual variations of the iliohypogastric nerve

Appearance	Total	Right	Left	*P*
Branching prior to entering the abdominal wall	198 (76.2%)	108 (76.6%)	90 (75.6%)	0.783
Entering the abdominal wall without branching	34 (13.1%)	16 (11.3%)	18 (15.1%)	0.191
Fusion with the ilioinguinal nerve	25 (9.6%)	16 (11.3%)	9 (7.6%)	0.076
Communicating branch with the subcostal nerve	3 (1.2%)	1 (0.7%)	2 (1.7%)	0.722

### Ilioinguinal nerve

**Fig. 2:**
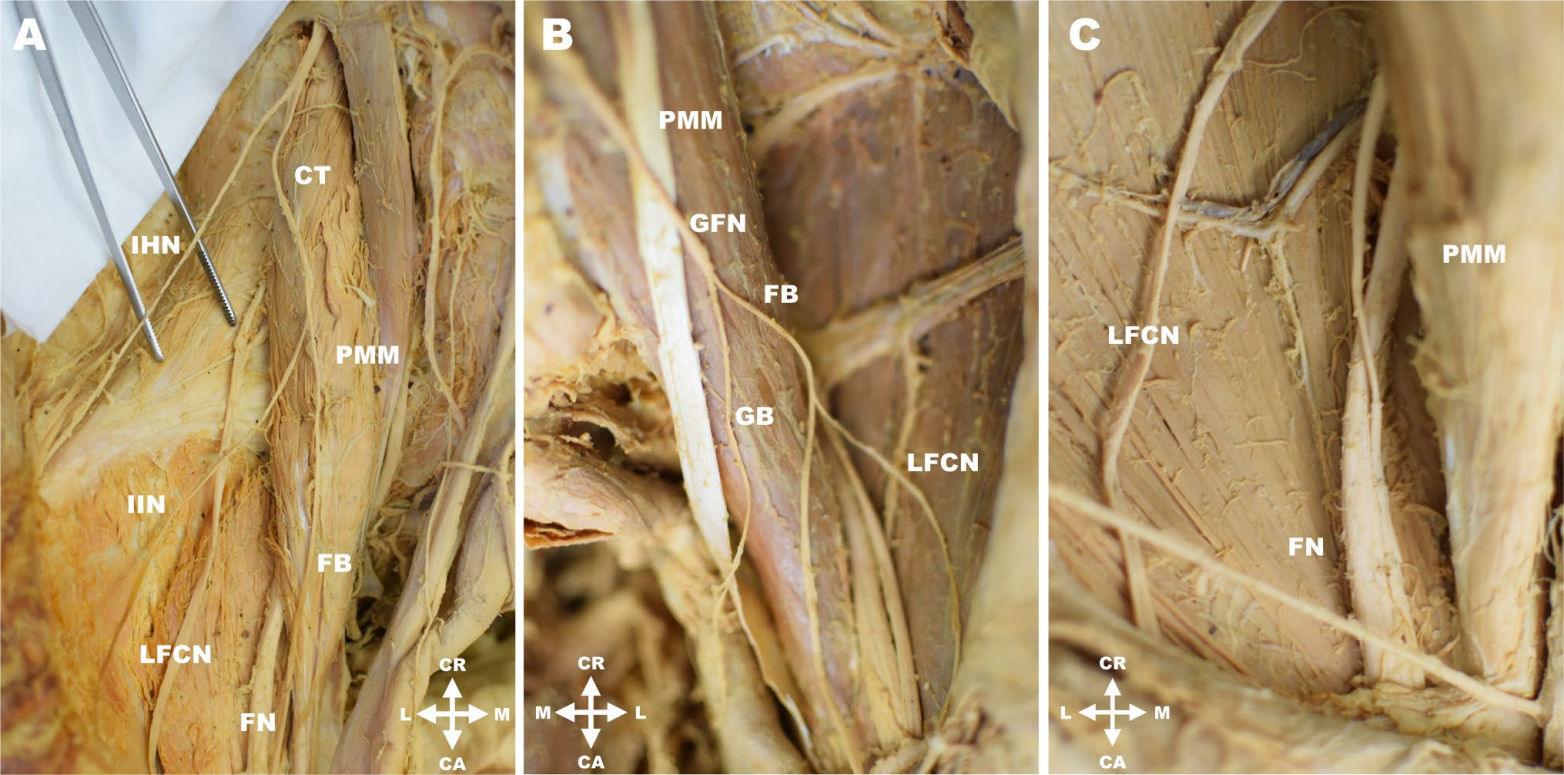
**A**. Right lumbar region showing a common origin of the iliohypogastric nerve (IHN) with common trunk (CT) for the ilioinguinal nerve (IIN) and the femoral branch of the genitofemoral nerve (FB). **B**. Left lumbar region showing the origin of the lateral femoral cutaneous nerve (LFCN) from a common trunk with the femoral branch of the genitofemoral nerve (FB). **C**. Right lumbar region with a low formation of the femoral nerve (FN). Legend: CA = caudal; CR = cranial; FB = femoral branch of genitofemoral nerve; FN = femoral nerve; GB = genital branch of genitofemoral nerve; GFN = genitofemoral nerve; IHN = iliohypogastric nerve; IIN = ilioinguinal nerve; L = lateral; LFCN = lateral femoral cutaneous nerve; M = medial; PMM = psoas major muscle

At the point of its exit from the psoas major muscle, the ilioinguinal nerve measured 1.8 ± 0.6 (1.0–3.1) mm in diameter. In 223 cases (85.8%) the nerve showed its usual latero-caudal course and ran deep into the dorsolateral abdominal wall as a single branch. As mentioned above, in 25 cases (9.6%) the ilioinguinal nerve left the psoas major muscle fused with the iliohypogastric nerve (Fig. 1B). Other variant morphology included seven cases (2.7%) where the ilioinguinal nerve originated from the lateral femoral cutaneous nerve just before the latter entered the muscular space and passed under the inguinal ligament, and four cases (1.5%) where the ilioinguinal nerve arose as a branch of the femoral nerve. Very rarely, there was one case (0.4%) where the ilioinguinal nerve had a common trunk with the femoral branch of the genitofemoral nerve, which arose from a common trunk with the iliohypogastric nerve (Fig. 2A). Detailed results are attached in Table 2.

**Table 2:** Proportions of the individual variations of the ilioinguinal nerve

Appearance	Total	Right	Left	*P*
Regular	223 (85.8%)	121 (85.8%)	102 (85.7%)	0.687
Fusion with the iliohypogastric nerve	25 (9.6%)	14 (9.9%)	11 (9.2%)	0.636
Origin from the lateral femoral cutaneous nerve	7 (2.7%)	2 (1.4%)	5 (4.2%)	0.124
Origin from the femoral nerve	4 (1.5%)	3 (2.1%)	1 (0.8%)	0.292
Origin from the femoral branch of the atypical genitofemoral nerve	1 (0.4%)	1 (0.7%)	0 (0%)	0.846

### Genitofemoral nerve

The genitofemoral nerve exited the psoas major muscle with a diameter of 2.0 ± 0.7 (0.5–4.1) mm. In 130 cases (50.0%), the nerve resembled its usual course, and divided into a femoral and a genital branch 4.6 ± 2.7 (3.8–7.0) cm distant from its exit from the psoas major muscle. Frequently, in 125 cases (48.1%), the femoral and genital branches left the psoas major muscle as two separate nerves with their typical course (Fig. 1C). As mentioned above, in one case (0.4%) the femoral branch arose from a common trunk with the ilioinguinal nerve (Fig. 2A). Besides these variations, the femoral branch arose from the lateral femoral cutaneous nerve prior to the entrance into the vascular space in one case (0.4%) (Fig. 1B), and in another three cases (1.2%) the femoral branch was found originating from the femoral nerve. Further results are shown in Table 3.

**Table 3:** Proportions of the individual variations of the genitofemoral nerve

Appearance	Total	Right	Left	*P*
Regular	130 (50.0%)	68 (48.2%)	62 (52.1%)	0.085
Exiting the psoas major muscle as two separate branches	125 (48.1%)	70 (49.6%)	55 (46.2%)	0.073
Origin of the femoral branch from the femoral nerve	3 (1.2%)	2 (1.4%)	1 (0.8%)	0.684
Origin of the femoral branch from the lateral femoral cutaneous nerve	1 (0.4%)	0 (0%)	1 (0.8%)	0.792
Origin of the femoral branch from the atypical ilioinguinal nerve	1 (0.4%)	1 (0.7%)	0 (0%)	0.846

### Lateral femoral cutaneous nerve

The lateral femoral cutaneous nerve emerged from the psoas major muscle with a diameter of 2.2 ± 0.6 (1.0–3.0) mm. The nerve featured its usual morphology in 247 cases (95.0%). In five cases (1.9%) a low formation of the lateral femoral cutaneous nerve was observed. Variant origin was noted in two cases, where the lateral femoral cutaneous nerve either arose from a common trunk with the femoral branch of the genitofemoral nerve (one case; 0.4%) (Fig. 1C) or directly from the femoral nerve (one case; 0.4%). An abnormal branching was encountered in six cases (2.3%) as the nerve featured high branching within the pelvic cavity prior to entering the muscular space and passing under the inguinal ligament (Fig. 1A, 1C). Detailed results are summarized in Table 4.

**Table 4:** Proportions of the individual variations of the lateral femoral cutaneous nerve

Appearance	Total	Right	Left	*P*
Regular	247 (95.0%)	133 (94.3%)	114 (95.8%)	0.731
Low formation	5 (1.9%)	4 (2.8%)	1 (0.8%)	0.285
High branching	6 (2.3%)	4 (2.8%)	2 (1.7%)	0.582
Origin from a common trunk with the femoral branch of the genitofemoral nerve	1 (0.4%)	0 (0%)	1 (0.8%)	0.792
Origin from the femoral nerve	1 (0.4%)	0 (0%)	1 (0.8%)	0.792

### Femoral nerve

**Fig. 3:**
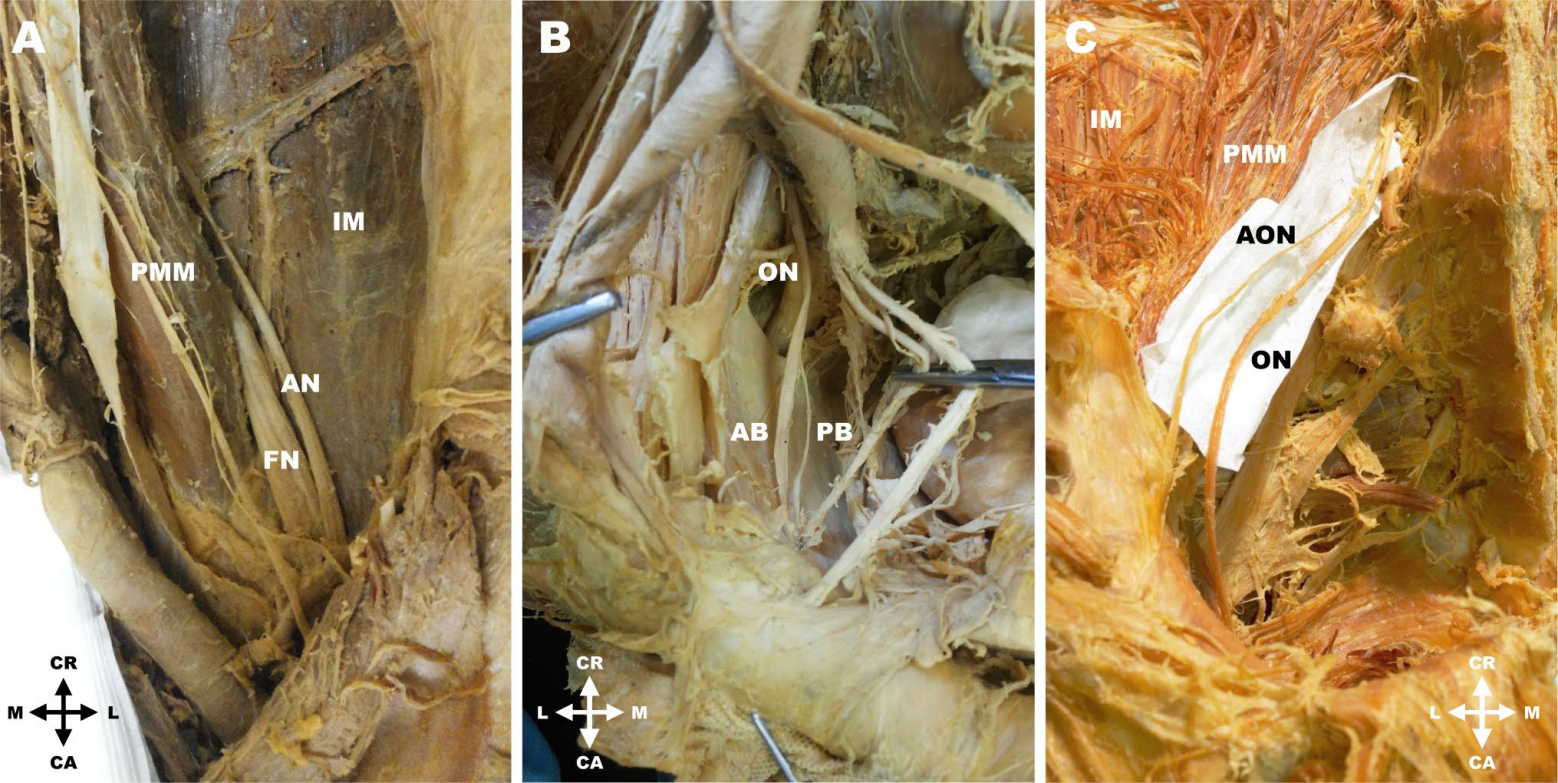
**A**. Left lumbar region with an accessory nerve supplying the sartorius muscle (AN). **B**. Right lumbar region with a high branching of the obturator nerve (ON) into anterior (AB) and posterior branches (PB). **C**. Right lumbar region with an accessory obturator nerve (AON). Legend: AB = anterior branch of obturator nerve; AN = accessory nerve; AON = accessory obturator nerve; CA = caudal; CR = cranial; FN = femoral nerve; IM = iliacus muscle; L = lateral; M = medial; PMM = psoas major muscle

At the point of its exit from the psoas major muscle the femoral nerve measured 6.3 ± 1.3 (4.0–8.2) mm. In the vast majority of cases (214 cases; 82.3%) the femoral nerve travelled typically according to the textbook description and entered the muscular space. In ten cases (3.8%) the femoral nerve featured a low formation outside of the psoas major muscle (Fig. 2C). In another 32 cases (12.3%) there was a smaller accessory nerve accompanying the proper femoral nerve that passed separately through the muscular space and variably innervated the muscles of the thigh (Fig. 3A). Also, high branching of the femoral nerve within the pelvic cavity was observed in four cases (1.5%). Detailed results are presented in Table 5.

**Table 5:** Proportions of the individual variations of the femoral nerve

Appearance	Total	Right	Left	*P*
Regular	214 (82.3%)	112 (79.4%)	102 (85.7%)	0.063
Accessory femoral nerve	32 (12.3%)	21 (14.9%)	11 (9.2%)	0.068
Low formation	10 (3.8%)	5 (3.5%)	5 (4.2%)	0.126
High branching	4 (1.5%)	3 (2.1%)	1 (0.8%)	0.292

### Obturator nerve

In all cases (100%) the obturator nerve did not show any variability at its exit from the psoas major muscle as it left the muscle medially (with a diameter of 2.6 ± 0.7; 2.1–4.5 mm) and descended toward the obturator canal. In 105 (40.4%) cases the obturator nerve showed high division into an anterior and a posterior branch before entering the obturator canal (Fig. 3B). In addition to the proper obturator nerve, an accessory obturator nerve was found in 24 cases (9.2%) (Fig. 3C). Detailed results are summarized in Table 6.

**Table 6:** Proportions of the individual variations of the obturator nerve

Appearance	Total	Right	Left	*P*
Regular	131 (50.4%)	61 (43.3%)	70 (58.8%)	0.075
High division	105 (40.4%)	63 (44.7%)	42 (35.3%)	0.183
Accessory obturator nerve	24 (9.2%)	17 (12.1%)	7 (5.9%)	0.248

## Discussion

The transpsoas approach to the lumbar spine provides effective access for treatment of several pathological conditions. However, to ensure safety and decrease postoperative morbidity, profound understanding of the surgical anatomy is required. As indicated in the presented study, nerves from the lumbar plexus, leaving the psoas major muscle, may appear in unexpected locations with atypical morphology. The enhanced knowledge presented herein provides an insight to the variational anatomy of the terminal branches of the lumbar plexus within the retroperitoneum, and may serve as guide for spine surgeons while interpreting the morphological appearance of these structure.

### Applied anatomy

**Fig. 4:**
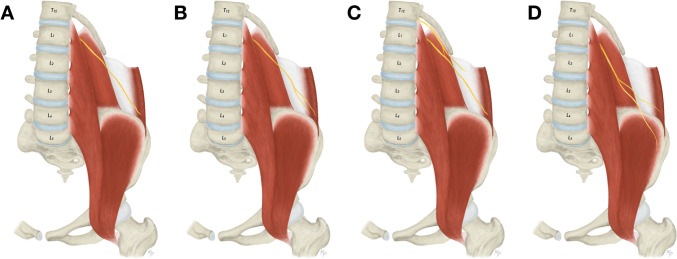
Variations of the iliohypogastric nerve anatomy. **A**. Usual appearance of the iliohypogastric nerve. **B**. Division of the iliohypogastric nerve prior to its entrance into the abdominal wall. **C**. Communicating branch between subcostal and iliohypogastric nerve. **D**. Common trunk for the iliohypogastric and ilioinguinal nerve with subsequent separation

**Fig. 5:**
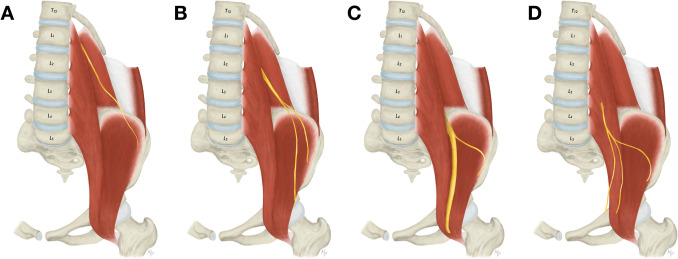
Variations of the ilioinguinal nerve anatomy. **A**. Usual appearance of the ilioinguinal nerve. **B**. Origin of the ilioinguinal nerve from a common trunk with the lateral femoral cutaneous nerve. **C**. Origin of the ilioinguinal nerve from the femoral nerve. **D**. Origin of the ilioinguinal nerve from a common trunk with the femoral branch of the genitofemoral nerve

During an access to the T12 and L1 vertebrae and interposed intervertebral disc, the subcostal, iliohypogastric, and ilioinguinal nerves are put at a risk of iatrogenic injury. According to the textbook descriptions, the subcostal nerve is the T12 anterior ramus and passes along (below) the twelfth rib to later pierce the aponeurosis of the transversus abdominis muscle [16]. However, from the anatomical point of view, the subcostal nerve is not deemed as an actual component of the lumbar plexus. The iliohypogastric nerve is described as emerging from the L1, eventually T12, anterior ramus and running on the anterior surface of the quadratus lumborum muscle (Fig. 4A). Close to the iliac crest it enters the dorsolateral abdominal wall through the transversus abdominis muscle as a single nerve. Based on our findings the iliohypogastric nerve frequently divides into two branches before entering the abdominal wall (76.2%) (Fig. 4B). Anomalous communicating branches can be identified between the subcostal and iliohypogastric nerves (1.2%) (Fig. 4C). Lastly, the ilioinguinal nerve originates predominantly from the L1 anterior ramus, and courses similarly within the abdominal cavity just caudal to the iliohypogastric nerve (Fig. 5A). The ilioinguinal nerve may not be detected at this level since it variably originates caudally from a common trunk with the lateral femoral cutaneous nerve (2.7%) (Fig. 5B), femoral nerve (1.5%) (Fig. 5C), or femoral branch of the genitofemoral nerve (0.4%) (Fig. 5D). Due to their close topographical relationship the iliohypogastric and ilioinguinal nerves may be frequently fused and form a single nerve (common trunk) at their exit from the psoas major muscle (9.6%) (Fig. 4D).

**Fig. 6:**
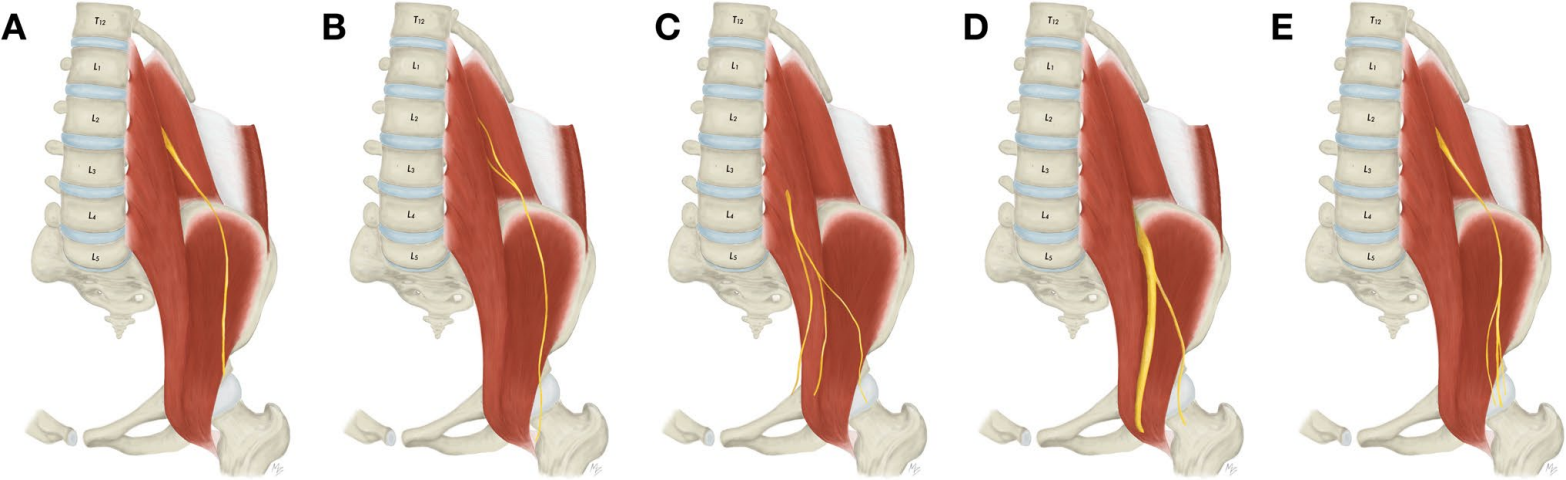
Variations of the lateral femoral cutaneous nerve anatomy **A**. Usual appearance of the lateral femoral cutaneous nerve. **B**. Low formation of the lateral femoral cutaneous nerve outside the psoas major muscle. **C**. Origin of the lateral femoral cutaneous nerve from a common trunk with the femoral branch of the genitofemoral nerve. **D**. Origin of the lateral femoral cutaneous nerve from the femoral nerve.** E**. High branching of the lateral femoral cutaneous nerve within the pelvic cavity

At the level of L1 and L2 all three above-mentioned nerves are encountered more laterally to the psoas major muscle. Apart from that, the lateral femoral cutaneous nerve is derived from the L2 anterior ramus with possible contributions from the L1 and L3 anterior rami [16]. It descends obliquely over the iliacus muscle towards the superior anterior iliac spine and passes underneath the inguinal ligament (Fig. 6A). Atypically, the lateral femoral cutaneous nerve may exhibit a low formation outside the psoas major muscle (1.9%) (Fig. 6B). Furthermore, it may originate more caudally than usual from a common trunk with the femoral branch of the genitofemoral nerve (0.4%) (Fig. 6C) or branching from the femoral nerve (0.4%) (Fig. 6D). Close to its exit from the pelvis, the lateral femoral cutaneous nerve may feature a high branching (2.3%) (Fig. 6E). Also, the L1 and L2 anterior rami give rise to the genitofemoral nerve, however, these rami usually unite more distally within the psoas major muscle.

While approaching the L2 and L3 vertebrae all previously described nerves can be found crossing over the quadratus lumborum muscle with the exception of the genitofemoral nerve, which is still hidden within the substance of the psoas major muscle. At this level, there is no nerve emerging from the psoas major muscle.

**Fig. 7:**
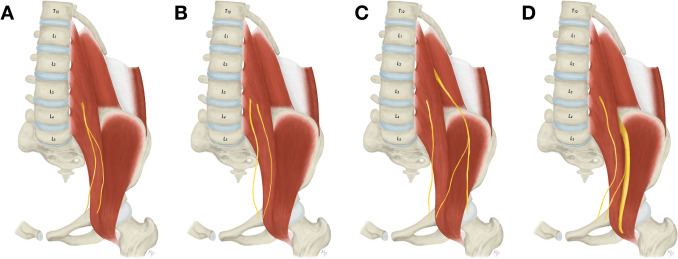
Variations of the genitofemoral nerve anatomy. **A**. Usual appearance of the genitofemoral nerve. **B**. Femoral and genital branches of the genitofemoral nerve exiting the psoas major muscle separately. **C**. Origin of the femoral branch of the genitofemoral nerve from a common trunk with the lateral femoral cutaneous nerve. **D**. Origin of the femoral branch of the genitofemoral nerve from the femoral nerve

The approach to the L3 and L4 vertebrae and the intervertebral disc located between them is connected with visualization of the genitofemoral nerve, which exits the psoas major muscle on its anterior surface at this level (Fig. 7A) [16]. It descends on the psoas major muscle and a few centimeters below the point of its exit it bifurcates into femoral and genital branches. The femoral branch then courses beneath the inguinal ligament, usually through the vascular space, while the genital branch runs beneath the falx inguinalis to enter the inguinal canal. As demonstrated in our study, the branches often leave the psoas major muscle separately (48.1%) (Fig. 7B). Therefore, identifying only one branch during surgery may mimic the proper genitofemoral nerve before its usual division. Moreover, the femoral branch may not be detected at all in this area because it variably originates more distally from a common trunk with the lateral femoral cutaneous nerve (0.4%) (Fig. 7C) or from the femoral nerve (1.2%) (Fig. 7D).

**Fig. 8:**
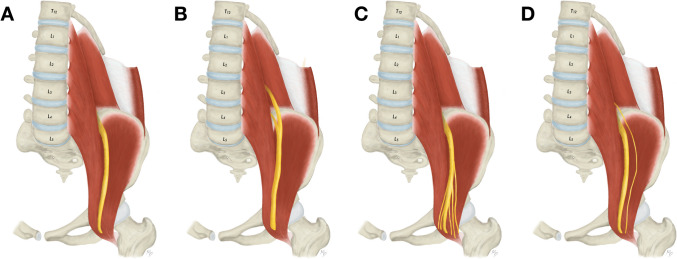
Variations of the femoral nerve anatomy. **A**. Usual appearance of the femoral nerve. **B**. Low formation of the femoral nerve outside the psoas major muscle. **C**. High branching of the femoral nerve within the pelvic cavity. **D**. Accessory nerve accompanying the proper femoral nerve

Lateral to the L4 and L5 vertebrae the femoral nerve leaves the psoas major muscle, which is formed by the L2 to L4 anterior rami (with variable addition of L1) intramuscularly [16]. Caudally, it descends between the lateral border of the psoas major muscle and the iliacus muscle, and passes underneath the inguinal ligament through the muscular space (Fig. 8A). More than one neural structure may be identified at this level, which are either low formation (3.8%) (Fig. 8B) or high branching (1.5%) of the femoral nerve (Fig. 8C), or an accessory nerve (12.3%) to the proper one that individually innervates some of the muscles of the thigh (Fig. 8D).

**Fig. 9:**
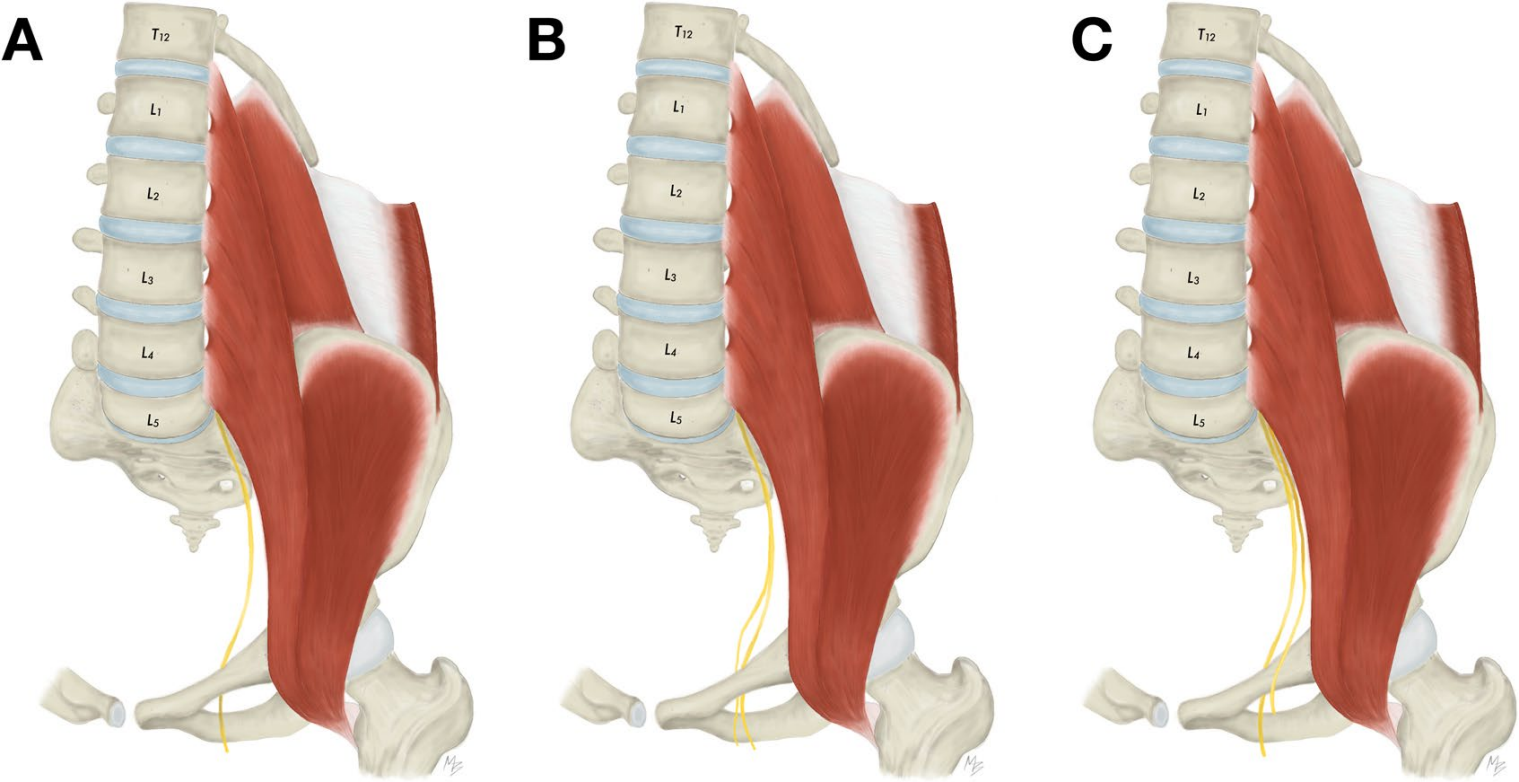
Variations of the obturator nerve anatomy. **A**. Usual appearance of the obturator nerve. **B**. High division of the obturator nerve. **C**. Accessory obturator nerve in addition to the proper obturator nerve

Although it is not routinely used, when accessing the L5 vertebra and S1 segment of the sacrum, all previously mentioned nerves are vulnerable both within the abdominal wall and inside the pelvic cavity adjacent to the psoas major muscle. Medially to the psoas major muscle the obturator nerve is located. Similarly to the femoral nerve, it is derived from the L2 to L4 anterior rami, which fuse within the belly of the psoas major muscle, and the definite nerve leaves the psoas major muscle from its medial aspect at the level of L5 [16]. The obturator nerve courses over the obturator internus muscle and enters the obturator canal (Fig. 9A). It is the only nerve joined by vessels (the obturator artery courses below the nerve, the obturator vein usually below the artery but sometimes variably in other positions) and covered from above by lymph nodes and vessels. Quite frequently, the obturator nerve branches into an anterior and a posterior branch prior to the entrance into the obturator canal (40.4%) (Fig. 9B). Variably, although relatively commonly, an accessory obturator nerve is present (9.2%) accompanying the proper obturator nerve (Fig. 9C). Nevertheless, each nerve shows different course as the accessory obturator nerve passes over the superior pubic ramus, instead of entering the obturator canal [16].

### Clinical implications

The rate of neurological complications following the lateral transpoas approach were declared in up to 33.6% for motor deficits, and up to 75% for sensory complications [1]. Furthermore, the severity of such complications include a spectrum from paresthesia to serious nerve palsies [17]. The causes of iatrogenic injury to the components of the lumbar plexus include a direct mechanical injury, resulting in traction, compression or laceration in cases of sharp instrument usage or an indirect ischemia [1].

Injury to the superior portion of the lumbar plexus, that includes the iliohypogastric, ilioinguinal and genitofemoral nerves, manifest as paresthesia, pain and numbness in the zone along the ilioinguinal line to the area below the anterior superior iliac spine. Motor dysfunction results in abdominal paresis, and the involvement of the ilioinguinal and genitofemoral nerves may be diagnosed upon absence of the cremaster reflex in males [1].

Involvement of the lateral femoral cutaneous nerve results in purely sensory impairment, called the meralgia paresthetica. This clinical syndrome is characterized as dysesthesia of the anterolateral thigh region [1].

Due to its extensive innervation the injury to the femoral nerve involves both motor and sensory deficits. Among the deficits in cutaneous innervation are especially the anteromedial thigh and medial crural dysethesias. Upon motor examination, weakness of hip flexion and external rotation, and knee extension, accompanied by a loss of patellar reflex, are highly suspicious for femoral nerve palsy [1].

Lastly, the obturator nerve carries both sensory and motor component. Sensory impairment refers to the sensation abnormalities of the medial aspect of the thigh, while the motor deficits presents as weakness of the hip adductors [1].

As shown in the results section, the nerves of the lumbar plexus may exhibit anomalous communications due to the presence of communicating branches or complete fusion of two adjacent nerves. This may, therefore, lead to unusual clinical symptomatology. For example, confusions may be caused by fusion of a sensory nerve with a motor branch, theoretically leading to a combined sensorimotor dysfunction.

### Comparison with anatomical studies

The anatomy of the lumbar plexus and its terminal branches has been investigated by several researchers. Nevertheless, most of the existing studies are rather focused only on particular nerves or investigated the formation of the lumbar plexus within the substance of the psoas major muscle [6, 8, 15]. Thus, the amount of complex studies is limited.

Communications between the iliohypogastric nerve and the subcostal nerve were present only in 1.2% of our cases. However, based on the data by Klaassen et al. [10] and Webber [18], there existed interconnecting rami in 22–26% of cases. Therefore, we suspect that contribution of the subcostal nerve to the iliohypogastric nerve is more frequent within psoas major muscle, than detected by us outside this muscle. Furthermore, the intimate relationship of the iliohypogastric and ilioinguinal nerves results in their frequent fusion, which was reported in 20–53% [6, 10].

Variations of the genitofemoral nerve are frequent and the high rate (20–42%) of separate origins of its two branches from the psoas major muscle was reported by several authors [6, 9, 11].

Unusual origin of the lateral femoral cutaneous nerve from the femoral nerve was found with an incidence of 7.5–8% [8, 18]. Most importantly, such origin was detected only in one of our specimens, therefore we believe, based on the methodology in previous studies, that the unusual origin from the femoral nerve occurs within the substance of the psoas major muscle. For this reason, we declare only one finding outside the psoas major muscle. According to a recent meta-analysis the lateral femoral cutaneous nerve exits pelvis as a single nerve in 79.1% [12]. Therefore, the early branching in 2.3% of our cases is less frequent than usual.

Accessory branches, in addition to the femoral nerve, are rather known as bifurcations of the femoral nerve within the substance of the iliopsoas muscle [18]. Since we did not further dissect the psoas major muscle to reveal the origin of the nerves, we could not use the same nomenclature. It is probable that the accessory nerve arose from the femoral nerve but in either case it is a separate nerve outside of the psoas major muscle within the pelvis that may be injured if not identified.

The obturator nerve showed early branching. Well-known variation is the presence of an accessory obturator nerve that is present in 13% of cases, according to the review by Turgut et al. [13].

The discrepancies between our findings and some previous studies may be also attributed to the demographic differences of studied populations. However, the most important factor may be the low sample size in the majority of previous studies, which may skew the actual prevalence rates, compared to our extensive observations, which represent, to the best of our knowledge, the largest anatomical cohort on the lumbar plexus reported in the modern scientific literature.

### Limitations and strengths

Since this study is purely focused on presenting potentially encountered neural variants during the transpsoas approach, the nerves of interest were only evaluated morphologically. Therefore, morphometric data, such as distances from constant structures or coordinates projected on the abdominal wall or vertebrae, were not collected. At the same time, only the extrapsoas portion of the lumbar plexus was studied, and, thus, the formation of the individual nerves within the substance of the psoas major muscle was omitted from our study. This includes overlooking the constitution of spinal nerves that form the lumbar plexus. Moreover, we did not assess the nerves with the use of microdissection to reveal the actual distribution of the neural fibers in the presence of any accessory communications. On the other hand, we present the largest recent study focusing on the anatomical variability of the terminal branches of the lumbar plexus. Such large sample-based studies are essential in order to state the actual frequency of the distinct variations.

## Conclusions

The results of this anatomical study revealed a high variability of the nerves of the lumbar plexus within the retroperitoneum. Most frequently, deviation from the textbook description was identified in the genitofemoral nerve. All other nerves of the lumbar plexus were far less variable. Noteworthy, anomalous communications, common trunks and variable origins of the individual nerves may lead to intraoperative confusions while identifying the nerves, and an atypical symptomatology may occur in case of their impairment. Furthermore, the presented study provides a compendious information source of the potentially encountered neural variations during retroperitoneal access to different segments of the lumbar spine. Thorough understanding of the surgically relevant variational anatomy presented herein may help minimize nerve injuries during the minimally invasive lateral transpsoas approach.

## Data Availability

Data that support the findings of this study are available upon reasonable request from the corresponding author.
